# Polyunsaturated Fatty Acid-Derived Lipid Mediators and T Cell Function

**DOI:** 10.3389/fimmu.2014.00075

**Published:** 2014-02-25

**Authors:** Anna Nicolaou, Claudio Mauro, Paula Urquhart, Federica Marelli-Berg

**Affiliations:** ^1^Manchester Pharmacy School, Faculty of Medical and Human Sciences, The University of Manchester, Manchester, UK; ^2^Centre for Biochemical Pharmacology, William Harvey Research Institute, Queen Mary University of London, London, UK

**Keywords:** T cells, polyunsaturated fatty acids, eicosanoids, prostaglandins, leukotrienes, cyclooxygenase, lipoxygenase, endocannabinoids

## Abstract

Fatty acids are involved in T cell biology both as nutrients important for energy production as well as signaling molecules. In particular, polyunsaturated fatty acids are known to exhibit a range of immunomodulatory properties that progress through T cell mediated events, although the molecular mechanisms of these actions have not yet been fully elucidated. Some of these immune activities are linked to polyunsaturated fatty acid-induced alteration of the composition of cellular membranes and the consequent changes in signaling pathways linked to membrane raft-associated proteins. However, significant aspects of the polyunsaturated fatty acid bioactivities are mediated through their transformation to specific lipid mediators, products of cyclooxygenase, lipoxygenase, or cytochrome P450 enzymatic reactions. Resulting bioactive metabolites including prostaglandins, leukotrienes, and endocannabinoids are produced by and/or act upon T leukocytes through cell surface receptors and have been shown to alter T cell activation and differentiation, proliferation, cytokine production, motility, and homing events. Detailed appreciation of the mode of action of these lipids presents opportunities for the design and development of therapeutic strategies aimed at regulating T cell function.

## Introduction

The regulation of energy metabolism is crucial to T cell-mediated immunity including activation, proliferation, and differentiation (1). Following recognition of antigen in the lymph nodes, naïve T lymphocytes undergo massive clonal expansion and differentiation, followed by a contraction or death phase, and the establishment and maintenance of immunological memory (2, 3). Before undergoing division, T cells activate biosynthetic pathways for the production of proteins, nucleic acids, lipids, carbohydrates, and other “building blocks” necessary for the generation of new cells. Following this stage, the metabolic machinery of T cells is reprogramed, switching from the β-oxidation of fatty acids in naive T cells to the glycolytic pathways in activated T cells (4–6).

Downstream of T cell receptor (TCR) signaling, phosphatidylinositol 3′-kinase (PI3K) leads to the activation of the serine–threonine kinase AKT, which promotes glucose metabolism by stimulating the localization of the glucose transporter Glut1 to the plasma membrane, and the activity of hexokinase and phosphofructokinase, two rate-limiting enzymes of the glycolytic pathway. Increased glycolytic flux enables activated T cells to generate ATP and, at the same time, efficiently utilize carbon sources in the form of amino acids and lipids for the biosynthesis of proteins and membranes necessary for the expansion phase that characterizes the immune response (7–11). AKT also controls the activation state of mammalian target of rapamycin (mTOR), a sensor of nutritional and energetic status in cells that promotes protein synthesis.

T cell activation also initiates distinct transcriptional programs, which determine their differentiation into functional subsets depending on the context [cytokines, prostaglandins (PG), and other extracellular signals] in which they were activated (12–14). These subsets define the characteristics of the immune response. Whereas CD8+ T cells differentiate into cytotoxic T lymphocytes that kill infected host cells, CD4+ T lymphocytes differentiate into either the Th1, Th2, or Th17 subset of helper T cells (effector T cells) that mediate appropriate immune responses or into induced regulatory T cells (iTreg cells) that suppress uncontrolled immune responses (12). There is evidence that the cytokine milieu in which T cells differentiate can influence their metabolic programing. A comparison of activated T cells responding to related cytokines IL-2 and IL-15 illustrates the differential regulation of T lymphocyte metabolism by distinct cytokine environments: IL-2 promotes elevated glucose metabolism and glycolysis, while IL-15 does not maintain this metabolic state and T cells responding to IL-15 are smaller with reduced nutrient uptake and glycolysis (15, 16).

After clearance of the infection, most clonally expanded and differentiated T cells undergo apoptosis (contraction phase). The surviving antigen-specific T cells (memory T cells) are responsible for enhanced immunity after re-exposure to the same pathogen. Of these various T cell subsets, the iTreg cells and memory T cells rely on lipid oxidation as a major source of energy, whereas cytotoxic T lymphocytes and effector T cells are characterized by high glycolytic activity (17–19).

Further to oxidation for energy production, fatty acids are involved in many other aspects of T cell biology. In particular, omega-3 polyunsaturated fatty acids (*n*-3 PUFA) are recognized as modulators of inflammation and immunity mediating their pleiotropic activity through regulation of gene expression, influencing signaling cascades, and altering the composition of the cellular membranes (20, 21). The latter has implications for the structure and function of the membrane, as well as a direct impact on the production of *n*-6 and *n*-3 PUFA-derived bioactive lipids including PG, leukotrienes (LT), resolvins (Rv), protectins (PD), endocannabinoids, and related congeners.

Although the immunomodulatory properties of PUFA have been known for many years, the molecular mechanisms underlying these properties are not fully understood. It has been shown that *n*-3 PUFA suppress antigen presentation, T cell activation and proliferation, and lower the expression of signature cytokines (21–27). Disappointingly, early studies using daily supplementation with foods rich in *n*-3 PUFA failed to show significant improvement in organ transplantation rejection (28, 29). However, recent reports indicate that administration of purified eicosapentaenoic acid (EPA; 20:5*n*-3) induces the differentiation of regulatory T cells through upregulation of peroxisome proliferator-activated receptor γ (PPARγ), a ligand-activated nuclear receptor that regulates lipid and glucose metabolism, leading to increased allograft survival (30, 31).

Following this direction, studies have explored the effect of cellular incorporation of the main *n*-3 PUFA, EPA, and docosahexaenoic acid (DHA; 22:6*n*-3). These fatty acids can alter the composition and molecular organization of membrane rafts with a consequent impact on the activity of raft-associated signaling proteins and related events. Examples include recruitment and activation of PLCγ and F-actin, impairing mitochondrial translocation necessary to maintain Ca^+^ signaling for NFκB and AP-1 activation and IL-2 secretion, and suppression of phosphatidylinositol-dependent actin remodeling, all linked to reduced CT4+ T cell activation [recently reviewed in Ref. (20)]. Importantly, many of the PUFA mediated activities are conveyed through their metabolites that tend to be produced and metabolized upon request, can act near the site of their synthesis or transported via circulation and in this way mediate systemic effects (autacoids). These families of potent mediators are intimately involved in inflammation and immunity, with pro- and/or anti-inflammatory, proliferative, and chemoattractive activities (21).

Overall, these new findings suggest that a better understanding of the molecular mechanism of action of PUFA may lead to the development of effective therapeutics. In this article, we will overview the current knowledge of the function and impact of eicosanoids and related metabolites, as well as that of endocannabinoids and their congeners on T cell function, and examine potential applications in biomedical research.

## PUFA-Derived Lipid Mediators: Biosynthesis and Metabolism

**Figure 1:**
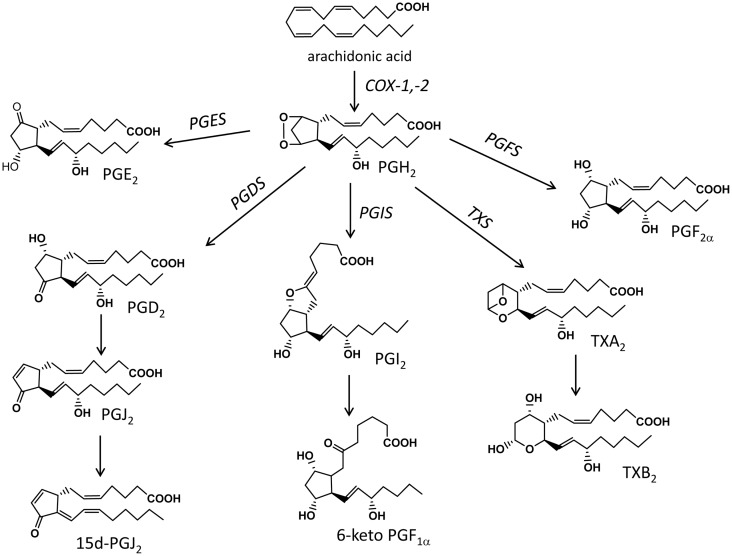
**Schematic representation of the main biochemical pathways that mediate the production of prostanoids**. COX, cyclooxygenase; PGES, prostaglandin E synthase; PGDS, prostaglandin D synthase; PGFS, prostaglandin F synthase; PGIS, prostacyclin synthase; TXS, thromboxane synthase.

**Figure 2:**
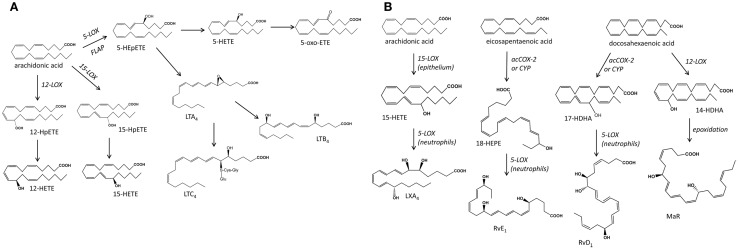
**Schematic representation of the main biochemical pathways that mediate the production of mono-hydroxy fatty acids and leukotrienes (A), and the poly-hydroxy fatty acids lipoxins, resolvins, and protectins (B), products of transcellular metabolism**. LOX, lipoxygenase; HETE, eicosatetraenoic acid; HEpETE, eicosaperoxytetraenoic acid; LT, leukotriene; acCOX-2, acetylated cyclooxygenase-2; CYP, cytochrome P450; LX, lipoxin; RvE, resolving series E; RvD, resolving series D; MaR, maresin.

**Figure 3:**
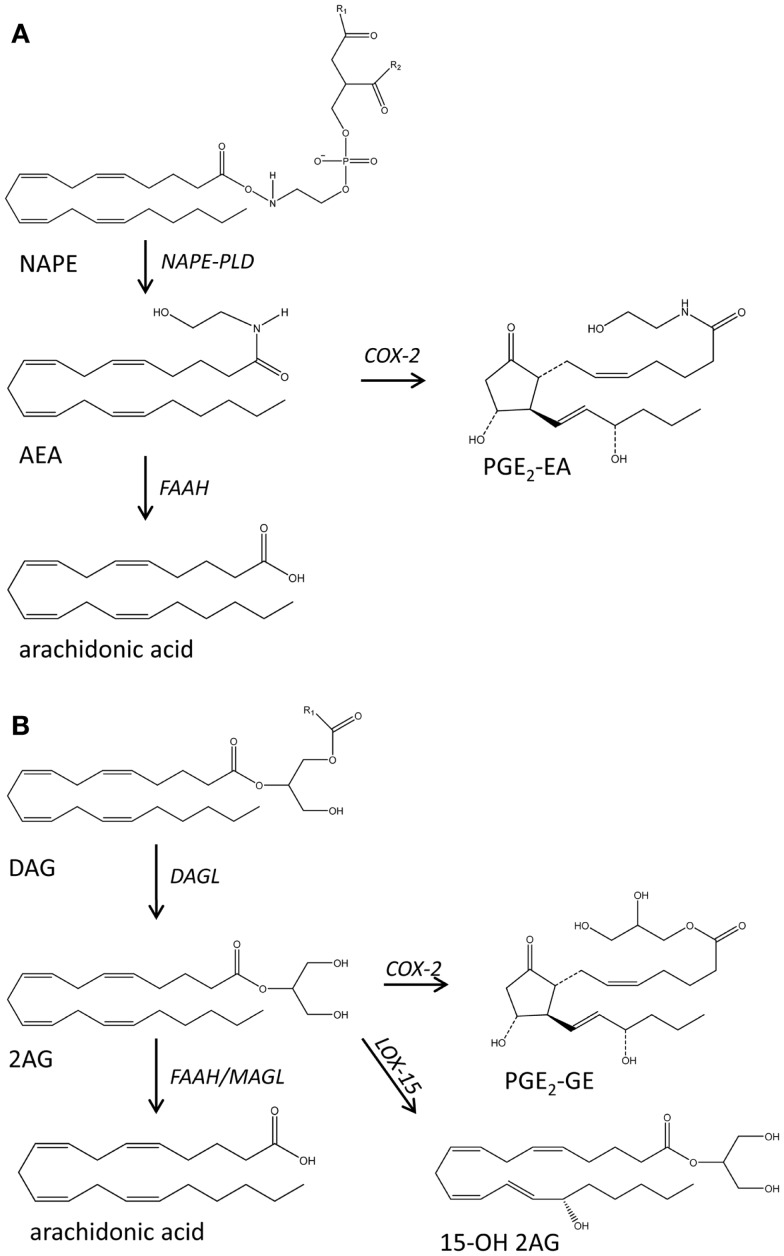
**Schematic representation of the main biochemical pathways that mediate the production of endocannabinoids anandamide (AEA) (A) and 2 arachidonoyl glycerol (2-AG) (B)**. DAG, diacylglycerol; DAGL, diacylglycerol lipase; FAAH, fatty acid amide hydrolase; 15OH 2AG, 15 hydroxy-2 arachidonoyl glycerol; MAGL, monoacylglycerol lipase; NAPE, *N*-acylated ethanolamine phospholipids; PGE_2_, PGE_2_ ethanolamide; PGE_2_-GE, PGE_2_-glyceryl ester.

The cellular membrane serves as a pool of PUFA available for further metabolism to various bioactive lipids. These potent autacoids act as local hormones and are produced upon request following the activation of signaling pathways or effect of environmental and other stimuli. The arachidonic acid (AA; C20:4*n*-3)-derived eicosanoids are some of the best known and studied bioactive lipids. The term “eicosanoids” is used to describe the bioactive derivatives of three fatty acids with 20-carbon acyl chains, namely: AA, EPA, and dihomo-gamma linolenic (DGLA; 20:3*n*-6). These metabolites, although mostly linked to inflammation, are also involved in cell migration, proliferation, chemotaxis, and immune reactions (32–34). Eicosanoids and related mediators derive from the activities of cyclooxygenases (COX), lipoxygenases (LOX), and cytochrome P450 (CYP) epoxygenases and mono-oxygenases (Figures 1 and 2) [reviewed in Ref. (35)]. The term “endocannabinoids” refers to endogenous lipids ligands of the cannabinoid receptors CB1 and CB2. These are also derivatives of AA, while other PUFA ethanolamides are now recognized as members of this family (36). Although endocannabinoids can be metabolized by COX and LOX, their precursor phospholipids and metabolism are different to eicosanoids (Figure 3).

### Cyclooxygenase-mediated formation of prostanoids

The eicosanoid cascade starts with the activation of phospholipases (PL), predominantly PLA_2_ but also PLD and diacylglycerol (DAG) lipase that release AA and other PUFA from the cellular membrane (35). The family of PLA_2_ comprises a large number of enzymes with distinct characteristics in terms of their activation, cellular localization, and substrate specificity (37). There is evidence for the presence of various PLA_2_ isoforms in primary T cells and the Jurkat T cell line, including cPLA_2_, sPLA_2_, and iPLA_2_ (38–42). Inducible isoforms of PLC and DAG lipase have also been identified in tumor and peripheral T lymphocytes (42, 43).

Free AA is then metabolized via the constitutive and inducible COX isoforms (COX-1 and -2, respectively) to the unstable endoperoxide PGH_2_ that is then transformed to PG, thromboxanes (TX), and prostacyclin (PGI_2_) via tissue specific terminal prostaglandin synthases (Figure 1); these COX-derived mediators belong to the family of eicosanoids and are collectively known as prostanoids. Apart from AA, prostanoids are formed from the other two 20-carbon containing PUFA, DGLA, and EPA, with the resulting metabolites having different activities and being considered less-inflammatory than the AA-derived ones (35, 44).

The exact profile of prostanoids is determined by the prevalence of specific synthases in the cell type or tissue of interest. PGE_2_ is produced by prostaglandin E synthase (PGES) that is found as membrane bound (mPGES-1 and -2) or cytosolic (cPGES). mPGES-1 is an inducible isoform and is frequently found co-expressed with COX-2 (45, 46). PGD_2_ is produced by the hematopoietic-type (H-PGDS) or the lipocalin-type (L-PGDS) synthases (47), while further non-enzymatic hydrolysis of PGD_2_ gives rise to the anti-inflammatory cyclopentanone PGs PGJ_2_ and 15d-PGJ_2_ (48, 49). PGF_2α_ is produced either directly from PGH_2_ via the prostaglandin F synthase (PGFS) or through further metabolism of PGE_2_ and PGD_2_ by PGE 9-ketoreductase and PGD 11-ketoreductase, respectively (50). Prostacyclin (PGI_2_) is produced via the prostacyclin synthase (PGIS) and is usually detected as its stable but inactive metabolite 6-keto-PGF_1α_ (51). Finally, thromboxane synthase (TXS) converts PGH_2_ to TXA_2_, an unstable prostanoid that is quickly hydrolyzed to the stable but inert metabolite TXB_2_ (51). The bioactivity of prostanoids is mediated through G protein-coupled receptors for PGE_2_, PGD_2_, PGF_2α_, PGI_2_, and TXA_2_, designated EP, DP, FP, IP, and TP, respectively. Pharmacological studies into their ligand-binding profiles and signal transduction pathways, and genetic analysis led to their classification into eight groups (EP1, EP2, EP3, EP4, DP1, FP, IP, and TP) although new developments have revealed the presence of a second PGD receptor, DP2, and the presence of heterodimers (52, 53). Overall, prostanoids are potent autacoids and their levels are controlled through enzymatic catabolism via dehydrogenations and reductions resulting in the formation of metabolites with significantly reduced bioactivities (e.g., 15-keto- and 13,14-dihydro-15-keto PGs) (54).

### Lipoxygenase-mediated production of leukotrienes and other hydroxy fatty acids

Lipoxygenases mediate the oxygenation of free fatty acids including AA and other PUFA. Their activities are commonly defined by their positional selectivity when they oxygenate AA and, following this system, the main mammalian LOX enzymes are defined as 5-, 12-, and 15-LOX. They catalyze the stereoselective insertion of OH in the *S* configuration, with the exception of a mammalian skin-specific enzyme 12*R*-LOX. The products of LOX reactions are unstable hydroperoxides that are then reduced to hydroxy acids (55–57) (Figure 2). 5-LOX acts in concert with 5-lipoxygenase activating protein (FLAP) to metabolize AA to 5*S*-hydroperoxyeicosatetraenoic acid (HPETE) that is further reduced to 5*S*-HETE or dehydrated to LTA_4_, an unstable epoxide containing a conjugated triene system characteristic of all LT. LTA_4_ can be metabolized to LTB_4_ or form the cysteinyl LT, LTC_4_, LTD_4_, LTE_4_ following conjugation with reduced glutathione (58). 5*S*-HETE can be also enzymatically reduced to the 5-oxo-eicosatetraenoic acid (5-oxo-ETE), a chemoattractant mediator (59). Mammalian 12- and 15-LOX isozymes oxygenate a range of PUFA, both free and esterified in membrane phospholipids and lipoproteins (57), forming a multitude of mono- and poly-hydroxy fatty acids: e.g., AA produces hydroxyeicosatetraenoic acids (HETE), EPA generates hydroxyeicosapentaenoic acids (HEPE), DHA produces docosanoids including hydroxydocosahexaenoic acids (HDHA), linoleic acid (LA; 18:2*n*-6) forms octadecanoids such as hydroxy octadecadienoic acids (HODE), DGLA forms hydroxyeicosatrienoic acids (HETrE), etc.

### Cytochrome P450 mediated fatty acid epoxides and their derivatives

Cytochrome P450 mono-oxygenases relevant to PUFA metabolism catalyze epoxygenations and mid-chain and omega-hydroxylations producing a range of LOX-like mono-hydroxy fatty acids (e.g., HETE, HEPE, HODE) although not necessarily of the *S* configuration [reviewed in Ref. (35)]. Interestingly, partially inhibited COX-2 (e.g., acetylated COX-2 following treatment with aspirin) can also generate LOX-like products with the OH group at *R* configuration, e.g., 15*R*-HETE from AA and 18*R-*HEPE from EPA (60). These metabolites are important in aspects of transcellular metabolism where sequential LOX/LOX or acetylated COX-2/LOX or CYP/LOX reactions involving more than one cell types are involved in the formation of multi-hydroxy fatty acid species. These include the lipoxins (LX) that are tri-hydroxytetraene-products of AA, and the di- and tri-hydroxy-PUFA termed Rv, PD, and maresins (MaR) that are derivatives of EPA and DHA. All these mediators are involved in inflammation and immunity exhibiting a range of protective roles (61–63).

### The endocannabinoids

The endocannabinoids anandamide (arachidonoyl ethanolamide, AEA) and 2-arachidonoyl glycerol (2AG) are derivatives of AA and act as endogenous ligands to the cannabinoid receptors CB1 and CB2 [reviewed in Ref. (36)]. This family of bioactive lipids has been extended to include other fatty acid ethanolamides and glycerols, while recent findings regarding their metabolism suggest a wider involvement in inflammation and immunity. The biochemical precursors of AEA and its congers are various *N*-acylated ethanolamine phospholipids (NAPE) that found in very low concentrations in the biological membranes and are hydrolyzed by NAPE-specific PLD or PLC-type lipases. 2AG production is mediated by PLC-diacylglycerol lipase. AEA and 2-AG can be deactivated via hydrolysis mediated by fatty acid amide hydrolases (FAAH) or can be metabolized by COX-2 to generated prostaglandin ethanolamides known as prostamides (e.g., PGE_2_-EA) and prostaglandin glyceryl esters (e.g. PGE_2_-GE) (Figure 3) (52). LOX isozymes can also metabolize these lipids although the prevalence and bioactivities of the resulting mediators remain to be explored.

## Eicosanoids and Related Mediators in T Cell Function/Biology

### Prostanoids

It is now recognized that resting and activated T cells express the COX-1/-2 system (64–68). Although the constitutive COX-1 is not affected during T cell activation, the inducible COX-2 is upregulated as has been shown in studies with CD4+ cells, Jurkat T cells and adaptive Tregs (66–69). To date, very little is known about the exact profile of prostanoids produced by T cells with only a few studies reporting the production of PGE_2_, PGD_2_ and its dehydration product 15d-PGJ_2_, as well as low levels of TXA_2_ (67, 68, 70). There is also very little information on the type of prostanoid synthases expressed in T cells, including evidence for H-PGDS and PGES in Tregs (67, 68). However, a number of studies have explored the role of PGE_2_, PGD_2_, PGI_2_, PGF_2α_, and TXA_2_ on various aspects of T cell function, showing that prostanoid-mediated effects process through receptors and related signaling pathways expressed in most T cell populations and subtypes. Interestingly, it has been shown that treatment with AA upregulates the CXCR3/1 inducible chemokine receptors expressed in CD4+ T cells and increases their chemotactic responses through a COX-related pathway (71), suggesting a potential role for this pathway in the regulation of T cell migration.

#### PGE_2_

Although considered to be a, primarily, pro-inflammatory eicosanoid, PGE_2_ can also mediate anti-inflammatory signals, and is a potent immunosuppressor (72). PGE_2_ is one of the best-studied bioactive lipids in T cell biology, exhibiting a multitude of effects. It is involved in the early stages of T cell development in the thymus, where it stimulates the differentiation of CD4+CD8+ thymocytes (73), while in later stages it regulates the development and balance of Th1, Th2, and Th17 subsets (74–76) and, overall, influences proliferation, differentiation, cytokine production, and apoptosis of mature T cells (14, 77–80). Interestingly, the activity of PGE_2_ on T cells appears to be concentration-dependent: while at low concentrations, it is involved in homeostatic events and inhibits the activation and differentiation of T lymphocytes, at high concentrations, PGE_2_ has the opposite effect, increasing T cell proliferation, and suppressing immune functions [recently reviewed in Ref. (81)]. For example, in ultraviolet radiation (UVR)-induced immunosuppression, impaired development of peripheral memory T cells can be attributed to UVR-induced PGE_2_ production (82).

Antigen presenting dendritic cells (DC) and macrophages secrete PGE_2_ and in this way can influence proliferation and differentiation of CD4+ and CD8+ cells, and direct the balance of Th1, Th2, and Th17 cell subtypes (14). PGE_2_ can also affect the maturation of DC and alter DC-produced cytokines, thus influencing the differentiation of T cell subtypes: for example, DC cells matured in the presence of PGE_2_ *in vitro* promote Th17 and inhibit Th1/Th2 polarization (78). PGE_2_ can also enhance the proliferation of T cells through the induction of costimulatory molecules OX40L, CD70, and 4-1BBL on DC (83), while other studies have reported that PGE_2_ inhibits the ability of DC to produce CCL19 and attract naive T cells (84). Interestingly, the ratio DC:T cells appears to be crucial in determining the overall immunogenic effect of PGE_2_: it has been reported that at high DC:T cell ratios, PGE_2_-maturated DC cells inhibit the proliferation of T cells, while, when this cell ratio is low, an enhanced T cell stimulation is observed (85). A dose-dependent effect has also been observed in the way PGE_2_ mediates the balance Th1 to Th2 subtypes: high levels of PGE_2_ suppress Th1 cell differentiation and polarization, shifting the immune response toward a Th2 phenotype (79). These observations have been confirmed *in vivo* using COX-2 inhibitors (e.g., celecoxib) and COX-2 knockout models demonstrating that when PGE_2_ production is reduced, an increase in Th1 responses is observed [reviewed in Ref. (81)]. The regulation of Th2 cells by PGE_2_ is likely to impact in Th2-mediated immune disorders such as atopic dermatitis and asthma (86, 87). Finally, when PGE_2_ is produced by activated macrophages it reduces T cell activation and proliferation; this in turn leads to a reduction in cytokine production and consequent reduced stimulation of macrophages in a negative feed-back loop (72).

*In vivo* work has elucidated the role of EP receptors in mediating PGE_2_ effects. PGE_2_ produced by DC in the lymph node acts through the EP1 receptor to promote the differentiation of naive T cells to Th1 cells (88). Studies on the BALB/c mice, a strain showing propensity to generate Th2 responses, have shown that Th2 cells express high levels of EP2 and that PGE_2_ signaling through this receptor protects Th2 cells against activation induced cell death (77). Furthermore, in a model of experimental autoimmune encephalomyelitis (EAE), PGE_2_ signaling through EP4 was shown to exert a dual role: promoting immune inflammation through Th1 cell differentiation and Th17 cell expansion during the induction phase. In contrast, during the effector phase of the disease, it attenuated the access of these pathogenic T cells to the brain by protecting the blood brain barrier (89, 90).

PGE_2_-induced effects mediated via the EP2/EP4 receptors are linked to cAMP concentration and related signaling (53). In cytotoxic T cells, PGE_2_ and other cAMP activators trigger increased concentration of cAMP and this interferes with the cytoskeleton function and terminates cytotoxic T cell secretion and adhesion (91). Dietary interventions with *n*-3 and *n*-6 PUFA can alter the cell membrane composition with consequent changes in the concentration of PGE_2_ produced, as well as the prevalence of the less-inflammatory PGE species, PGE_1_ and PGE_3_ (44). Although frequently cited as anti-inflammatory, these species do not always appear to be different in their immunomodulatory properties: for example, studies have shown that both PGE_2_ and PGE_1_ suppress mitogen-induced blastogenesis in T cells, an effect confirmed with experiments using indomethacin, a non-specific COX inhibitor (92).

PGE_2_ ethanolamide appears to be also involved in the motility of T cells (93, 94) and recent work using imaging has identified PGE_2_ as an antagonist of the T cell migration stop signal (95). This activity was shown to be subset specific, with Th migration in response to IL-2 inhibited at 10–100 ng/ml PGE_2_ *in vitro*, although, in the same experimental conditions, the migration of cytotoxic T cells was not affected (96, 97). PGE_2_ has also been suggested to inhibit the transendothelial migration of T cells through increased calcium and cAMP concentrations (98, 99). In rats, PGE_2_ was found to inhibit the migration of T cells across the microvascular retinal endothelial cells although it did not affect the expression of adhesion molecules on either endothelial or T cells (100). However, PGE_2_ at nanomolar to micromolar concentrations elicited migration of T cells *in vitro* and increased secretion matrix metalloproteinases (MMP); although MMP inhibitors suppressed the transmigration, the inhibition did not affect the PGE_2_-initiated T cell motility (101). Finally, overexpression of COX-2 in a mouse breast cancer model increased the recruitment of Tregs in the tumor, an effect mediated via EP2 and EP4 receptors (102).

#### PGD_2_ and 15d-PGJ_2_

PGD_2_ is considered an immunomodulatory prostaglandin and some of its cyclopentanone PG metabolites, such as 15-deoxy-Δ^12,14^-PGJ_2_ (15d-PGJ_2_), are endowed with anti-inflammatory activities (49, 103). Production of PGD_2_ has been detected in Th2 cells and this was linked to expression of H-PGDS, while L-PGDS has not been identified in any T cell subtype (67, 104, 105). The downstream product of PGD_2_ dehydration, 15d-PGJ_2_, has also been detected in human T cell cultures (67).

PGD_2_ mediates its effects through two receptors DP1 and DP2, the latter better known as chemoattractant receptor-homologous molecule expressed on Th2 cells (CRTH2). DP1 belongs to the prostanoid family of receptors, signals through cAMP and has been detected in Th1, Th2, and CD8+ cells (106). DP2/CRTH2 has little similarity to prostanoid receptors and belongs to the cytokine receptor family; it signals through increased calcium and inhibition of cAMP and has been found to be preferentially expressed by activated Th2 cells mediating their recruitment and motility (106, 107). While PGD_2_ can signal through either receptor, findings to date indicate that 15d-PGJ_2_ activates only DP2 (103). It has been suggested that PGH_2_ may also be an agonist of DP2 (108). PGD_2_ and 15d-PGJ_2_ are also agonists of PPARγ and can induce differentiation of fibroblasts to adipocytes; this has been shown in the case of Grave’s disease where it was reported that activated T cells drive fibroblast differentiation in ocular tissue through production of PGD_2_ and 15d-PGJ_2_, implying that T cell infiltrates can influence fat deposition in other tissues (67).

PGD_2_ can mediate different effects depending on the target receptor and related signaling events (109). DP1 can induce differentiation of Th2, whilst DP2/CRTH2 is mostly involved in their recruitment, although the two receptors may exert opposing effects, as examined in an animal model of contact hypersensitivity where DP2/CRTH2 appeared to mediate inflammatory events while DP1 was inhibitory (110). Furthermore, both receptors have been reported involved in T cell proliferation, and DP1 has been suggested to promote T cell apoptosis and downregulate immune responses, while DP2 has been reported to delay Th2 apoptosis (111). A potentially anti-inflammatory protective effect of 15-dPGJ_2_ in pregnancy has been attributed to its suppression of Th1 response and promotion of Th2 immunity through DP2 (112).

Activation of Th2 cells by PGD_2_ is thought to occur predominantly through DP2/CRTH2 with concomitant increase in the production of cytokines and pro-inflammatory proteins (106, 113–115). PGD_2_ binding to this receptor is also very important for CD4+ T cell trafficking and motility (116, 117). When produced at high concentrations by mast cells, as seen in allergic inflammation, there is a consequent activation and recruitment of Th2 cells toward the PGD_2_ producing sites (118, 119). Activated T cells can also produce PGD_2_ and this may promote further accumulation of Th2 in the inflamed tissue (107, 116).

Finally, PGD_2_ has been shown to affect the maturation of monocyte derived DC impacting on their ability to stimulate naive T cells and favoring their differentiation toward Th2 cells (120, 121). Interestingly, age related increase in PGD_2_ levels have been associated with decreased DC migration and reduced T cell responses in a mouse model of respiratory infections, suggesting that inhibition of PGD_2_ functions may be an effective therapeutic approach (122).

#### PGF_2α_

To date, there is very limited information on the contribution of this vasoactive prostaglandin on T cell function. There are no reports on the production of PGF_2α_ or expression of the relevant synthases on T lymphocytes. Early work exploring the involvement on PG on T cell locomotion considered the involvement of PGF_2α_ but this was not supported by the resulting data (93). However, a recent report on allergic lung inflammation presents evidence for the contribution of PGF_2α_ in Th17 cell differentiation, an autocrine effect mediated through cell surface FP receptors (123).

#### PGI_2_

PGI_2_ is best known as an inhibitor of platelet aggregation and potent vasodilator, while recent finding has shown its involvement in immune regulation with particular importance in airway inflammation. The IP receptor is expressed in a number of immune cells in the lung, including T lymphocytes of the Th1 and Th2 lineage (124, 125). However, there is very little information on the actual production of PGI_2_ by T cells with only some indirect evidence for possible transcellular biosynthesis operating between platelets and lymphocytes, and some recent work showing PGIS mRNA in an animal model of contact hypersensitivity (125, 126).

Studies in various models suggest that PGI_2_ is involved in regulating the balance of Th1 and Th2 responses, as well as promoting Th17 cell differentiation (13, 127). Work in a mouse model of asthma has shown that PGI_2_ produced by endothelial cells and signaling through the IP receptor prevents the recruitment of Th2 in the airways (128). However, a mouse model of contact hypersensitivity shows that in cutaneous disease PGI_2_-IP signaling raises intracellular cAMP concentration and promotes Th1 differentiation (125). Furthermore, PGI_2_ increased the ratio of IL-23/IL-12 leading to differentiation of Th17 cells and exacerbation of EAE in mice (129). Finally, the anti-inflammatory effect of PGI_2_ has been explored through analogs that reduced the production of pro-inflammatory cytokines and chemokines by DC, increased the production of anti-inflammatory IL-10, and inhibited their ability to stimulate CD4+ T cell proliferation (124).

#### TXA_2_

Although production of TXA_2_ by T cells has been reported, albeit at very low levels, the expression of the relevant synthase has not yet been shown (70, 130). However, the TP receptor has been found in a range of T cell populations and a polymorphism identified in Th2 cells has been linked to aspirin-exacerbated respiratory disease (130–133). Work with human lymphocytes suggested that TXA_2_ is involved in the inhibition of T cell proliferation and related cytokine production (134). Following production of TXA_2_ by DC, stimulation in TP expression was observed and this appeared to be involved in the random movement of naive but not memory T cells, suggesting that TXA_2_ can mediate DC–T cell interactions (130).

### Leukotrienes, hydroxy fatty acids, lipoxins, resolvins, and protectins

Lipoxygenase isoforms identified in various T cell populations include 5-, 12-, and 15-LOX (135–138). Although some early studies suggested that externally provided AA could inhibit 5-LOX, recent reports have indicated that provision of substrate may be necessary for the synthesis of LTs (135, 139). There is evidence that 5-HETE, LTA_4_ and LTB_4_, and the cysteinyl LT LTC_4_, LTD_4_, and LTE_4_, are produced by human and animal primary T cells and cell lines (43, 135, 138, 139). Furthermore, the presence of 5-LOX and 12/15-LOX would suggest the production of hydroperoxy- and hydroxy-PUFA by T cells. Nevertheless, there are not many studies examining the formation of such mediators and the majority of relevant reports focus on the effect of 12- and 15-HETE, LX, resolvins, and PD on T cell function.

#### LTB_4_

The main activity attributed to LTB_4_ is chemotaxis, a property mediated through the high affinity receptor BLT1 that is expressed in many CD4+ and CD8+ T cell subtypes (140–143). BLT1 is also important for homing events, as it enables the adhesion of T cells to epithelial cells, and appears of particular importance for the recruitment and direction of T cells to the airways in asthma (141, 144). Blockade of LTB_4_/BLT1 pathway has also been shown to improve CD8+ T cell mediated colitis (145). Finally, LTB_4_ appears involved in Th17 cell differentiation, Th1 and Th2 proliferation, and cytokine production (146–149).

#### LTC_4_, LTD_4_, LTE_4_

The cysteinyl LT specific receptors CysLT1 and CysLT2 have been found to be expressed by peripheral blood T cells (150). Interestingly, it has been reported that resting Th2 cells display higher expression of the CysLT1 receptor compared to Th1 or activated Th2 cells, suggesting its involvement in Th2 cell differentiation (151, 152). Accordingly, in the presence of PGD_2_, LTD_4_ and LTE_4_ have been shown to enhance Th2 cell activation and cytokine production, in a more than additive effect (153).

Furthermore, LTC_4_ appears to induce T cell proliferation (154), while LTC_4_-maturated DC appear to stimulate CD4+ responses and induce cytotoxic T cells *in vitro* without concomitant recruitment of Tregs (155).

#### 5-HETE and 5-oxo-ETE

Oxidative stress appears to stimulate the metabolism of 5-HETE to 5-oxo-ETE in peripheral blood lymphocytes, although the role of this lipid mediator in T cell function is not clear (156, 157).

#### 12-, 15-HETE

12-HETE has been involved in T cell function, with particular relevance to allergic disease. Although 12(S)-HETE is a neutrophil chemoattractant it does not appear to have a similar effect on T cells. Work on skin-derived lymphocytes involved in psoriasis has shown that 12(*R*)-HETE, a 12*R*-LOX product found in psoriatic skin, has modest chemotactic properties for T cells but is less potent than LTB_4_ (158, 159). Furthermore, it has been shown that inhibition of 12/15-LOX enhanced the production of Th2 cytokines and attenuated the development of allergic inflammation in a mouse model of allergic lung disease, whilst delivery of 12(S)-HETE had the opposite effect (136). Increased levels of 12-HETE were also associated with metabolic changes in T cells leading to development of autoimmune disease (137).

It has been reported that 15-HETE regulates T cell division and displays anti-proliferative effects on a leukemia T cell line (160–162). Metabolism of 15-HETE through β-oxidation has been observed in blood T cells leading to the hypothesis that the resulting β-hydroxy acids and their oxidized and decarboxylated products may play a role in T cell biology (163). 15-LOX metabolites have also been involved in Th1 responses in a mouse model of Th1 allergic inflammation induced by double-stranded RNA (164).

#### Lipoxins

Although not directly produced by T cells, LXA4 has been shown to interact with the LTB_4_ receptor expressed in T cells (165, 166). Aspirin-triggered LXA_4_ and LXB_4_, and stable analogs, inhibited TNFα production by human peripheral blood T cells suggesting the involvement of these metabolites in T cell mediated inflammation (167). Finally, LXA_4_ appears to be involved in Treg-mediated tumor protection through the induction of myeloid suppressor cells, as shown in a murine liver cancer model (168).

#### Resolvins and protectins

These products of EPA and DHA are formed through transcellular metabolism and some of their anti-inflammatory and pro-resolution effects are mediated through their effects on T cells. It has been reported that PD1 is formed by Th2-skewed peripheral blood mononuclear cells and appeared to block T cell migration, inhibit TNFα and IFγ secretion, and promote apoptosis *in vivo* (169). Reduction of CD4+ and CD8+ T cell infiltrates and CD4+ T cell-produced cytokines was also observed in a mouse model of DNFB-induced atopic dermatitis treated with RvE1 (170). Furthermore, RvE1-treated bone marrow-derived DC appear to induce apoptosis of T cells, and it has been suggested that instead of migrating to the lymph nodes they remain on the inflammatory sites targeting the infiltrating effector T cells (171). RvE1 has also been shown to reduce the influx of Th1 and Th17 cells in the cornea of a mouse model of stromal keratitis, a virally induced immunopathological disease; it has been suggested that this may have contributed to a significant reduction in lesions observed (172).

### Endocannabinoids and congeners

The endocannabinoid system is considered an important regulator of the immune response with AEA, 2AG, and related enzymes and receptors being involved in T cell function (173–176). Production of AEA and 2AG have been shown in human T lymphocytes (177, 178), while the receptors CB1 and CB2 have been identified in primary T cells and T cell lines where their expression is stimulated upon activation (179, 180). In particular, the CB2 receptor has been shown to mediate the inhibition of mixed lymphocyte reactions by cannabinoids and is of interest for the development of novel chemotherapeutic approaches to prolong graft survival (181). Furthermore, CB2 has been suggested as an important factor for the formation of T cell subsets including splenic memory CD4+ cells and natural killer T cells (182). Interestingly, a common CB2 gene polymorphism has been linked to reduced immune modulation by endocannabinoids and may be a risk factor for autoimmune disorders (183). Finally, FAAH and monoacylglycerol lipase (MAGL) are also present in human T lymphocytes (179). FAAH appears to play a protective role controlling the levels of AEA in pregnancy as well as immune-mediated liver inflammation (178, 184).

#### AEA and congeners

Work with activated primary human T lymphocytes has shown that AEA can suppress T cell proliferation and cytokine release in a CB2-dependent manner, without exerting cytotoxic effects (185, 186). However, other studies suggested that AEA inhibits T cell proliferation and induces apoptosis through a mechanism that may not be receptor mediated but most probably related to lipid rafts (187, 188).

The immunosuppressive effect of AEA extends to Th17 cell and this is of particular interest for the development of immunotherapeutic approaches (186). Endogenous AEA or inhibition of FAAH leading to increased AEA levels, were effective in reducing cytokine levels, decreased liver injury, and increased numbers of Treg cells in a murine model of immune-mediated liver inflammation (184). AEA inhibited the migration of CD8+ T cells in a collagen-based migration assay, again through the CB2 receptor (189). However, a study evaluating the direct anti-cancer potential of AEA, reported no effect on lymphocyte proliferation or Treg generation or cytokine production (190). In contrast, other studies have reported proinflammatory effects by AEA. In a mouse model of atherosclerosis, reduced levels of FAAH that resulted in increased AEA and its congeners, palmitoyl- and oleoyl-ethanolamide, were accompanied by reduced CD4+FoxP3+ regulatory T cells, suggesting a pro-inflammatory effect on the overall immune response (191). In addition, AEA appears to promote Th1 immunity as shown in a model of sensitization where it was reported to induce DC activation and IFNγ production (192). Finally, a recent study with bimatoprost suggested that this prostamide can induce calcium signaling in human T cells (193).

#### 2AG

The chemotactic properties of 2AG are also mediated through the CB2 receptor and this has been shown in various immune cells including migration of splenocytes (194), homing of B cells (195), and motility of human natural killer cells (196). When this potential was assessed in activated T lymphocytes, it was reported that although 2AG did not induce T cell migration, it inhibited migratory responses toward the chemokine CXCL12, suggesting a possible regulatory role in T cell migration (179). Furthermore, 2AG can act as DC chemoattractant and indirectly shift the memory response toward a Th1 phenotype in a CB2-mediated fashion (197). 2AG can also suppress IL-2 production in Jurkat cells through PPAR-γ activation and independently of CB1 and CB2-mediated signaling (198). The contribution of a COX-2 metabolite of 2AG has also been considered by recent reports confirming that the 15-deoxy-delta (1)(2),(1)(4)-PGJ_2_-glycerol ester (15d-PGJ_2_-GE) is a PPAR-γ ligand that suppresses IL-2 production in activated Jurkat cells (111, 199).

## Concluding Remarks

While current evidence support a key role for PUFA-derived bioactive lipids in the regulation of T cell immunity (Table 1), the complexity of their biological properties and the lack of a comprehensive understanding of their exact contribution to different stages of the immune response hinders the identification of mediators of interest either as markers or as target compounds for drug development. In general, it appears that lipid mediators regulate T helper cell polarization into Th1/Th2 and Th17 cells, a key event in many immune-mediated diseases. Despite the molecular mechanisms for this effect and the regulatory role of these lipids on other T cell functions have yet to be explored, an extensive number of studies in mice and humans underscore their therapeutic potential.

**Table 1:** **Summary of the main immunoregulatory roles of bioactive lipid mediators related to T cell function and biology**.

Lipid mediator	Receptor	Effect on T cells	Reference
PGE_2_	EP1, EP2/EP4	Differentiation	(73, 78, 79, 81, 88)
	EP2/EP4	Proliferation	(72, 81, 83, 85)
	EP2/EP4	Cytokine production	(72, 77–80)
	EP2/EP4	Apoptosis	(77, 91)
	EP2/EP4	Motility of T cells	(93–95)
	EP2/EP4	Treg recruitment	(102)
	EP2/EP4	Th1, Th2, Th17 balance	(14, 74–76, 78, 79)
PGD_2_	DP1	Differentiation of Th2; T cell apoptosis	(110, 120, 121)
	DP1, DP2	Recruitment, proliferation of Th2	(111)
	DP2	Activation, cytokine production, trafficking, and motility of Th2	(106, 107, 113–119)
15d-PGJ_2_	DP2	Suppression of Th1 and promotion of Th2	(103, 112)
		DC–T cell interaction	(120, 121)
PGF_2α_	FP	Th17 differentiation	(123)
PGI_2_	IP	Th1/Th2 balance	(13)
		Th1, Th17 differentiation	(125, 127, 129)
TXA_2_	TP	Inhibition of T cell proliferation	(134)
		Mediation of DC-T cell interactions	(130)
LTB_4_	BLT1	Homing	(141, 144)
		Differentiation, proliferation, and cytokine production	(146–149)
CysLTD_4_	CysLT1	Th2 differentiation	(151–153)
CysLTE_4_			
12-HETE		Weak T cell chemotaxis	(158, 159)
		Metabolic changes	(137)
15-HETE		Proliferation	(160–162)
		Th1 responses	(164)
LXA4	BLT1	Cytokine production	(167)
AEA	CB2	Suppression of Th1 and Th17 proliferation and cytokine release	(185, 186)
	–	Inhibition of proliferation; apoptosis via membrane rafts	(187, 188)
		Increased Tregs	(184)
2AG	CB2	T cell migration	(179)
		Suppression of cytokine production via PPAR-γ	(189)

This concept is supported by the large number of studies using their precursor fatty acids. Of particular importance is the focus on *n*-3 PUFA that have been explored as anti-inflammatory and immune-protective agents for a range of diseases and relevant experimental models including psoriasis, rheumatoid arthritis, and atherosclerosis (32, 33, 200). A recent study has shown that dietary *n*-3 PUFA favorably modulate intestinal inflammation in part by downregulating pathogenic T cell responses (201). The Fat-1 mouse, a genetic model that synthesizes long-chain *n*-3 PUFA *de novo*, was shown to be relatively resistant to colitis induction due to a reduced differentiation of Th17 cells and related cytokines (202). The immunoregulatory potential of a number of fatty acids has been reported over the years including that of DGLA and GLA (203), stearidonic acid (204) as well as various CLA mixtures used for inflammatory bowel syndrome and human Crohn’s disease (205). Parenteral administration of fatty acids has been shown to ameliorate disease via immunomodulatory effect in a model of rat sepsis (206), A randomized study in patients awaiting carotid endarterectomy showed that *n*-3 PUFA ethyl esters are incorporated into advanced atherosclerotic plaques and higher plaque EPA is associated with decreased plaque inflammation and T cell infiltration, and increased stability following dietary supplementation with EPA (207).

Furthermore, altering the profile of lipid mediators to strengthen the responses of T cells may be of value to cancer immunotherapy and could result in the development of potent and/or less toxic therapeutics. For example, it is well-documented that most tumors express PGE_2_ and this can contribute to immune suppression (103, 208). Pharmacological inhibition of PGE_2_ via non-steroidal anti-inflammatory drugs or EP receptor agonists could be supported or even replaced by systemic administration of EPA, precursor of the less potent eicosanoid PGE_3_ and the anti-inflammatory resolving series E (RvE) that can tone down the PGE_2_-mediated effects. Finally, a large number of other investigations have reported that immunonutrition with fatty acids leads to amelioration of a variety of immune-mediated disease by targeting T cell function. Examples include studies showing that the use of *n*-3 PUFA can improve lung injury and sepsis in animal models, and reduce infectious complications in patients undergoing major surgery and following severe trauma (209–211), while other reports draw attention to the contribution of fatty acids and their mediators in vaccine-induced immunity in infants, the prevention of experimental autoimmune encephalomyelitis through inhibition of Th1/Th17 differentiation by DHA, EPA-mediated protection of cardiac allografts, and amelioration of contact dermatitis following DHA and AA supplements (212–215).

Overall, there is a strong case for further developing therapeutic approaches based on the use of bioactive lipids as immunomodulators. The unmet challenge to fully exploit their therapeutic potential will be to unravel the circuits and molecular mechanisms by which these powerful mediators impact on T cell-mediated immunity.

## Conflict of Interest Statement

The authors declare that the research was conducted in the absence of any commercial or financial relationships that could be construed as a potential conflict of interest.
